# Prevalence and associated factors of depression, anxiety, and stress among flood-affected women in Bangladesh: a case study following 2024 flash flood

**DOI:** 10.3389/fpubh.2026.1830406

**Published:** 2026-07-09

**Authors:** Khawla Saeed Al Hattawi, Md. Mostafizur Rahman, Edris Alam, Ifta Alam Shobuj, Md. Tanvir Hossain

**Affiliations:** 1 Faculty of Resilience, Rabdan Academy, Abu Dhabi, United Arab Emirates; 2 Faculty of Arts and Social Sciences, Department of Disaster Management and Resilience, Bangladesh University of Professionals, Dhaka, Bangladesh; 3 Department of Geography and Environmental Studies, University of Chittagong, Chittagong, Bangladesh; 4 Sociology Discipline, Social Science School, Khulna University, Khulna, Bangladesh

**Keywords:** Bangladesh, depression, anxiety, and stress, disaster vulnerability, flash flood, women’s mental health

## Abstract

Floods are frequent climate-related disasters in Bangladesh, threatening physical safety, livelihoods, and mental health. Women are particularly vulnerable due to gendered roles, economic dependence, and caregiving duties. However, research on women’s mental health outcomes following recent flash floods is limited. A community-based cross-sectional study was conducted among 504 women aged 18 years and above in Fulgazi and Sonagazi upazilas of Feni District, Bangladesh, following the 2024 flash flood. Participants were recruited purposively from flood-affected communities, and data were collected at the beginning of 2025. Data were collected via face-to-face interviews using a structured questionnaire, and mental health was assessed using the DASS-21. Severe depression (71.1%), anxiety (48.8%), and stress (60.6%) were reported. Women aged 18–25 years reported lower depression and anxiety, while those aged over 55 years showed higher anxiety. Engagement in income-generating activities was associated with lower levels of depression, anxiety, and stress. Perceived social life satisfaction was strongly associated with satisfaction, whereas dissatisfaction was associated with higher psychological distress. Chronic disease status was significantly associated with mental health, with women having chronic conditions reporting higher anxiety, and those experiencing flood-related illness showing elevated depression, anxiety, and stress. Flood-related exposures also played a key role; the perceived lack of safety was associated with higher anxiety. Access to socioeconomic support during the flood was associated with lower anxiety and stress, while access to safe drinking water reduced anxiety. Food security and evacuation experiences also influenced outcomes, with the absence of food scarcity linked to lower depression and non-evacuation associated with higher depression. High levels of depression, anxiety, and stress were observed among women in flood-affected areas of Feni, Bangladesh, highlighting the need for gender-sensitive mental health support in disaster-prone settings.

## Introduction

Floods are among the most frequent and destructive climate-related disasters worldwide, affecting hundreds of millions of people annually and causing substantial social, economic, environmental, and public health consequences (1–3). As climate change intensifies the frequency and severity of extreme weather events, floods are increasingly recognized not only as environmental disasters but also as major public health emergencies. In addition to immediate threats such as injury, mortality, displacement, and infectious disease outbreaks, floods can generate substantial and long-lasting psychological consequences that undermine individual well-being and community resilience. Increasing evidence indicates that flood survivors frequently experience elevated levels of depression, anxiety, stress, and post-traumatic stress disorder (PTSD) that may persist long after floodwaters recede (4–8). These mental health consequences are often exacerbated by ongoing socioeconomic stressors such as livelihood loss, housing damage, displacement, financial insecurity, and disruption of social networks.

Bangladesh is one of the world’s most flood-prone countries due to its location within the Ganges–Brahmaputra–Meghna delta, low-lying topography, high population density, and exposure to monsoon rainfall and cyclonic events (1, 2, 9, 10). Recurrent floods affect millions of people each year and are expected to intensify under changing climatic conditions (11–13). In August 2024, a severe flash flood struck Feni District and surrounding regions, causing widespread inundation, displacement, disruption of livelihoods, and damage to essential infrastructure and services (14, 15). The sudden onset and widespread impacts of this event created substantial challenges for affected communities and raised concerns regarding both immediate and long-term mental health consequences.

The psychological consequences of disasters have become an increasingly important area of public health research. Flood exposure can trigger emotional distress through multiple pathways, including direct exposure to hazardous events, loss of property and income, disruption of daily routines, uncertainty about recovery, and concerns regarding future disasters. Previous studies have consistently reported elevated rates of depression, anxiety, stress, and PTSD among flood-affected populations across diverse geographic settings (7, 16). These psychological effects may persist for months or even years after the disaster, particularly when recovery resources are limited and socioeconomic vulnerabilities remain unresolved.

Emerging evidence indicates that flood-affected populations in Bangladesh exhibit a high prevalence of psychological distress. For instance, studies following the 2024 flash flood have documented widespread symptoms of depression, anxiety, and stress among affected residents, with women and other vulnerable groups showing disproportionately higher levels of distress (7, 17). Female gender, lower educational attainment, chronic health conditions, economic insecurity, and limited access to resources have been identified as important factors associated with adverse mental health outcomes in disaster settings, highlighting the complex interaction between social vulnerability and disaster exposure.

Women frequently experience disproportionate psychological impacts following disasters due to gender-specific social, economic, and caregiving responsibilities. The Gender and Disaster Vulnerability Framework suggests that pre-existing gender inequalities shape women’s exposure, sensitivity, and adaptive capacity during disasters. Women often have fewer economic resources, reduced decision-making power, greater caregiving obligations, and more limited access to disaster-related information and support services compared with men. These structural inequalities may increase vulnerability to psychological distress during and after disaster events. Global research consistently demonstrates a gendered pattern in mental health responses, with women generally reporting higher rates of depression and anxiety than men in post-disaster settings (18–20). In Bangladesh, women commonly bear primary responsibility for childcare, elder care, household management, food preparation, and water collection. During flood events, these responsibilities often intensify while access to resources becomes increasingly constrained, placing women at greater risk of emotional exhaustion, anxiety, and psychological distress.

Despite the growing body of literature on disaster-related mental health in Bangladesh, important knowledge gaps remain. Existing studies have primarily focused on general populations, mixed-gender samples, or broad disaster contexts. Consequently, limited empirical evidence exists regarding the mental health status of women affected by the 2024 flash flood in Feni District, particularly when examined through a comprehensive set of social, economic, health-related, and disaster-exposure variables. Furthermore, little is known about how these intersecting vulnerabilities collectively influence depression, anxiety, and stress among women during the post-flood recovery period. Addressing this gap is essential for developing evidence-based, gender-sensitive mental health interventions and disaster recovery strategies in flood-prone communities.

This study is informed by the Conservation of Resources (COR) Theory (21), the Social Vulnerability Framework (22), and the Gender and Disaster Vulnerability Framework (23). According to COR Theory, psychological distress arises when individuals experience actual or threatened loss of valued resources, including income, housing, health, social support, and personal security. The Social Vulnerability Framework emphasizes that disaster impacts are shaped by pre-existing socioeconomic inequalities and differential access to resources. The Gender and Disaster Vulnerability Framework further highlights how gendered social roles and structural inequalities influence women’s exposure to hazards, coping capacities, and recovery outcomes. Together, these theoretical perspectives suggest that disaster exposure, resource loss, health conditions, and social support interact to shape post-disaster mental health outcomes among women.

Based on these theoretical perspectives, a conceptual framework was developed in which disaster exposure factors (e.g., flood duration, perceived safety, early warning dissemination, and evacuation experience), resource-related conditions (e.g., income-generating activities, socioeconomic support, food security, and access to safe drinking water), health-related factors (e.g., chronic disease and flood-related illness), and psychosocial characteristics (e.g., social life satisfaction and household composition) collectively influence depression, anxiety, and stress among flood-affected women. Within this framework, flood exposure may disrupt livelihoods and living conditions, resource loss may intensify insecurity and uncertainty, and caregiving responsibilities may either buffer or exacerbate psychological distress depending on available support systems.

Accordingly, the study had two primary objectives:

- To determine the prevalence and severity of depression, anxiety, and stress among women affected by the 2024 flash flood in Feni District, Bangladesh.
- To identify the sociodemographic, social, economic, health-related, and disaster-exposure factors associated with depression, anxiety, and stress among flood-affected women.

In line with previous evidence, it was anticipated that adverse conditions such as unemployment, lower social satisfaction, chronic illness, perceived lack of safety, food scarcity, illness during the flood, and limited access to support would be associated with higher levels of psychological distress. Conversely, access to resources and stronger social connectedness were expected to be associated with lower levels of distress. However, given the complex and context-dependent nature of disaster experiences, these relationships were examined empirically rather than assumed. By employing the Depression, Anxiety, and Stress Scale-21 (DASS-21) (24) and simultaneously examining a broad range of social, economic, health-related, and disaster-specific factors, this study provides a gender-focused and context-specific assessment of mental health outcomes following the 2024 Feni flash flood. The findings are expected to contribute to a more comprehensive understanding of women’s mental health vulnerabilities in disaster settings and to inform gender-responsive disaster preparedness, recovery planning, and mental health interventions in Bangladesh and other flood-prone regions.

## Methods

### Study design and setting

**Figure 1:**
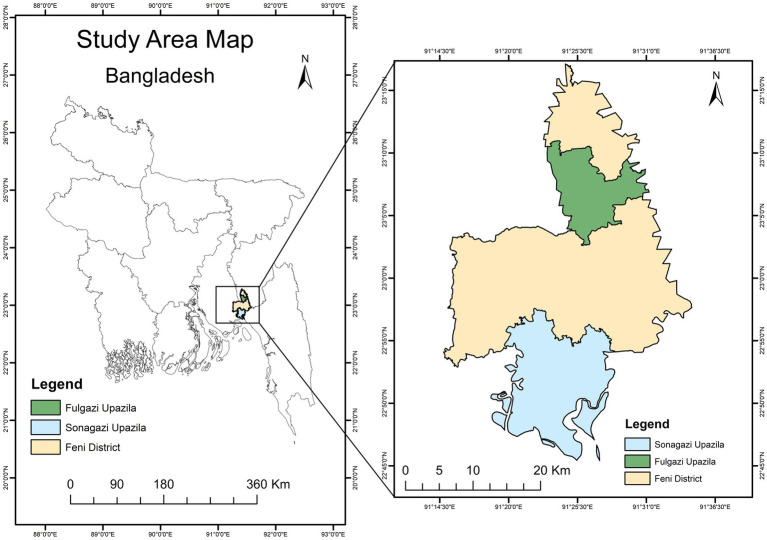
Study area.

A community-based cross-sectional study was carried out to examine the mental health impacts on women affected by the 2024 The study was carried out in Fulgazi and Sonagazi upazilas (Figure 1), two of the most severely affected areas during the flood. The flood was triggered by heavy monsoon rainfall combined with upstream water flow, leading to widespread flooding, displacement, and disruption of livelihoods and essential services. Data were collected from January to February 2025 over six weeks, approximately 4–5 months after the August 2024 flash flood event. This interval was considered appropriate for assessing post-disaster mental health outcomes while allowing sufficient time for immediate emergency conditions to stabilize.

Feni, previously a subdivision of Noakhali District, became a district (25). It is located between 22°44′ and 23°17′ north latitude and 91°15′ and 91°35′ east longitude. The district borders Comilla District and India to the north, India and Chattogram District to the east, Chattogram and Noakhali Districts to the south, and Noakhali District to the west.

Fulgazi Upazila is situated in the northern part of Feni District, covering approximately 102.19 square kilometers (26). It is bordered by Tripura, India, to the east and features fertile floodplains crossed by rivers such as the Muhuri, Selonia, and Kahuya (27). Fulgazi has a population of about 119,558 and a literacy rate of about 60% (27). The upazila has been prone to flooding due to its low-lying topography and proximity to major rivers. The 1998 floods, for example, caused significant damage to settlements and agriculture in the area (28). The 2024 floods exacerbated this vulnerability, inundating over 40 villages and affecting thousands of residents (14, 29).

Sonagazi Upazila, covering around 284.89 square kilometers, has a population of roughly 262,547 (27). It borders Fulgazi and Chhagalnaiya, forming part of a region highly susceptible to flooding. Sonagazi has also experienced severe flooding in recent years. In August 2024, reports indicated that approximately 350,000 people in Sonagazi, Fulgazi, and Chhagalnaiya were affected by rising waters, severely disrupting daily life and infrastructure (14, 29, 30). Flooding in the area typically results in submerged roads and the loss of access to essential services.

### Study population and eligibility criteria

Women were eligible to participate if they were aged 18 years or older, resided in the selected study areas during the August 2024 flash flood, directly experienced one or more flood-related impacts (including household inundation, loss of household income, evacuation to a shelter or alternative location, disruption of routine daily activities, food scarcity, difficulty accessing safe drinking water, or other substantial flood-related hardships), were permanent residents of the study area, and provided informed consent. Women were excluded if they were younger than 18 years, had not directly experienced the 2024 flash flood, were temporary residents or visitors during the flood period, had communication difficulties that prevented participation in an interview, or were severely ill, medically unstable, or otherwise unable to complete the interview at the time of data collection. The exclusion of severely ill women may have introduced selection bias, as some of the most vulnerable individuals, who may have experienced greater psychological distress, were not represented in the final sample. Consequently, the findings should be interpreted with consideration of this potential limitation.

### Sampling technique

The required sample size was estimated using the single population proportion formula for cross-sectional studies:

n=Z2p(1−p)/d2

Assuming a prevalence of 50% (to maximize sample size), a 95% confidence level (Z = 1.96), and a margin of error of 5%, the minimum sample size was 384 participants. After allowing for an anticipated 10% non-response rate, the required sample size increased to approximately 423 participants. A total of 504 women were ultimately recruited, thereby exceeding the minimum sample requirement and improving statistical precision.

A purposive sampling strategy was employed because the study specifically targeted women who had been affected by the 2024 flash flood. A household listing of flood-affected households was first prepared with the assistance of local administrative representatives, community leaders, and field verification. This listing served as the sampling frame for participant recruitment.

Field investigators visited identified households sequentially. If more than one eligible adult woman resided in the same household, one respondent was selected using a simple random selection procedure to avoid clustering of responses within households. If the selected woman was unavailable, up to two repeat visits were made before classifying the household as a non-response. A total of 530 eligible women were approached. Of these, 504 agreed to participate and completed the interview, yielding a response rate of 95.1%.

### Data collection instrument and procedures

Data were collected using a structured interviewer-administered questionnaire comprising sections on sociodemographic characteristics, flood-related experiences and exposure, health-related conditions, social and economic factors, and mental health outcomes. Depression, anxiety, and stress were assessed using the DASS-21 (24). The questionnaire was initially developed in English and subsequently translated into Bangla. To ensure conceptual equivalence, linguistic accuracy, and contextual appropriateness, the translated version was reviewed by experts in public health and disaster research. Prior to the main survey, a pilot study was conducted among approximately 5% of the estimated sample in a flood-affected community outside the selected study areas. Feedback obtained from the pilot testing was used to refine the questionnaire by improving the clarity, wording, sequencing, and cultural relevance of the items. Data collected during the pilot study were excluded from the final analysis.

Data collection was conducted by trained enumerators with prior experience in field-based public health research. Enumerators received a discussion program covering study objectives, interview techniques, administration of the DASS-21 instrument, ethical considerations, informed consent procedures, confidentiality requirements, and management of sensitive mental health questions. Mock interviews and practical exercises were conducted during the discussion. Face-to-face interviews were conducted in Bangla and required approximately 25–35 min to complete. Supervisors reviewed completed questionnaires daily to identify inconsistencies, missing responses, and data-entry errors. Regular field monitoring and verification procedures were implemented throughout data collection to ensure data quality.

### Measurement of mental health outcomes

Depression, anxiety, and stress were assessed using the validated Bengali version of the DASS-21 (31). The DASS-21 consists of 21 items divided into three seven-item subscales measuring depression, anxiety, and stress (24). It is important to note that the DASS-21 is a screening and symptom-severity instrument and does not provide clinical diagnoses of depressive disorders, anxiety disorders, or stress-related disorders. Instead, the instrument measures the severity of self-reported symptoms experienced during the preceding week. Each item was scored on a four-point Likert scale ranging from 0 (“Did not apply to me at all”) to 3 (“Applied to me very much or most of the time”). Subscale scores were summed and multiplied by two according to standard DASS-21 scoring procedures. Severity categories were classified using established DASS-21 guidelines (Table 1).

**Table 1:** Cut-off values for the DASS-21’s labels for depression, anxiety, and stress (24).

Severity label	Depression	Anxiety	Stress
Normal	0–9	0–7	0–14
Mild	10–13	8–9	15–18
Moderate	14–20	10–14	19–25
Severe	21–27	15–19	26–33
Extremely severe	28+	20+	34+

Internal consistency was evaluated using McDonald’s omega coefficient. Omega values were 0.69 for depression, 0.82 for anxiety, and 0.85 for stress. While the omega coefficient for depression was slightly below the commonly preferred threshold of 0.70, it remained within the lower acceptable range for exploratory research and was considered adequate for the present study.

### Contextual and disaster-related variables

In addition to mental health outcomes, several contextual and disaster-related indicators were assessed. Because most of these variables were measured using single-item questions, they should be interpreted as contextual indicators rather than validated psychological constructs. Examples included social life satisfaction (measured by asking, “How satisfied are you with your current social life?,” with options ranging from least satisfied, satisfied, to very satisfied), perceived safety from future floods (“How safe do you feel in your current place of residence from future flooding?,” with options for moderately safe or unsafe), socioeconomic support (“Did you receive any financial, material, or livelihood-related assistance during or immediately after the 2024 flash flood?,” answered yes/no), food scarcity (“Did your household experience shortages of food during the flood period?,” answered yes/no), and access to safe drinking water (“Did you have access to safe drinking water during the flood?,” answered yes/no). Notably, the variable ‘mental health impact due to the flood’ was excluded from inferential analyses because all respondents answered affirmatively, resulting in zero statistical variation. Furthermore, this variable conceptually overlapped with the DASS-21 outcome measures, thereby providing limited analytical value.

### Data analysis

Data were entered, cleaned, and analyzed using statistical software. We employed Python (version 3.12; Beaverton, OR 97008, USA) and R (version 4.2.2) (32, 33) for data management. Descriptive statistics were used to summarize participants’ sociodemographic characteristics, disaster-related experiences, and mental health outcomes. Continuous variables were described using means and standard deviations (SD), whereas categorical variables were summarized using frequencies and percentages. The selection of explanatory variables was guided primarily by the study’s conceptual framework and evidence from previous disaster mental health literature. Sociodemographic, health-related, social, economic, and disaster-exposure variables identified as theoretically relevant were therefore considered in the regression analyses rather than being selected solely based on statistical significance in univariable analyses. Depression, anxiety, and stress scores derived from the DASS-21 were treated as continuous outcome variables. Associations between explanatory variables and each mental health outcome were examined using multiple linear regression models. Regression coefficients (*β*), corresponding 95% confidence intervals (95% CI), R^2^, and adjusted R^2^ were reported to quantify the magnitude and direction of associations. Prior to model estimation, diagnostic procedures were conducted to assess linear regression assumptions, including multicollinearity, linearity, normality of residuals, and homoscedasticity. Although some evidence of heteroscedasticity was observed, the degree of violation was not considered sufficient to invalidate the overall interpretation of the regression models. Therefore, the findings should be interpreted with appropriate caution. Several explanatory variable categories contained relatively small numbers of respondents, including younger age groups, participants reporting chronic disease, those engaged in income-generating work, and those exposed to longer flood durations. Consequently, estimates associated with these categories may be less stable and should be interpreted cautiously. Although a large proportion of respondents reported severe psychological symptoms, DASS-21 scores demonstrated sufficient variability across the full range of observed values and were therefore analyzed as continuous outcomes using linear regression. This approach is consistent with previous studies examining disaster-related mental health outcomes using DASS-21 subscale scores.

### Ethical issue

This study was conducted as part of an approved research project (Ref. No. KUECC-2022/06/16) by the Ethical Clearance Committee of Khulna University, Khulna, Bangladesh. The research followed all ethical principles outlined in the Declaration of Helsinki and its subsequent revisions (34). Written informed consent was obtained from all participants prior to data collection. For respondents who were unable to read or write, the consent form was read aloud in Bangla by the interviewer. Participants were allowed to ask questions and receive clarification regarding study procedures. Confidentiality and anonymity were maintained throughout the study, and participants were informed of their right to withdraw at any time without penalty.

## Results

### Sociodemographic characteristics of respondents

**Figure 2:**
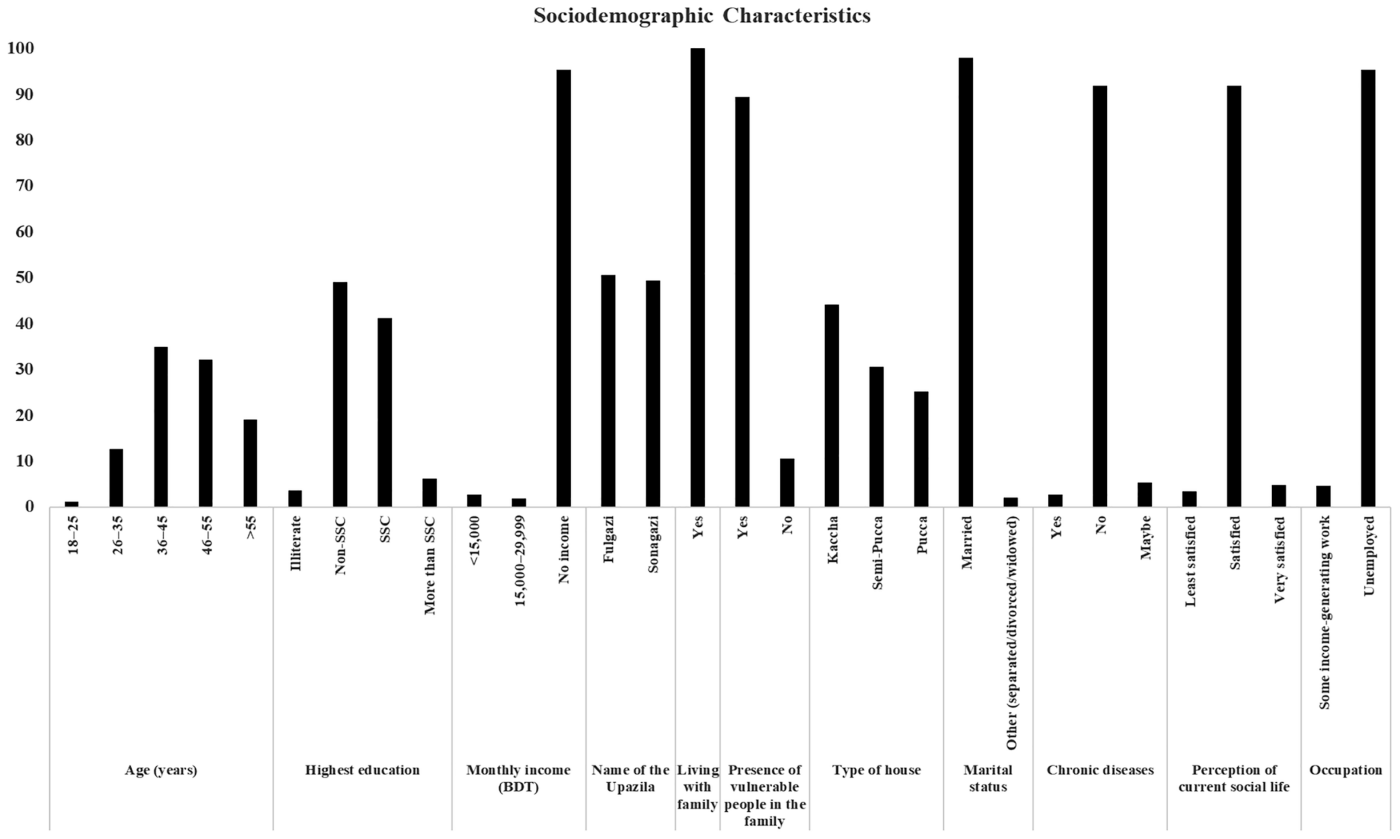
Sociodemographic characteristics of the respondents (*n* = 504).

Figure 2 presents the sociodemographic characteristics of the 504 women respondents affected by the 2024 flash flood in Feni District, Bangladesh. Most respondents were aged 36–45 years (34.92%) or 46–55 years (32.14%), while 19.05% were older than 55 years. Nearly half (49.01%) had educational attainment below the Secondary School Certificate (SSC) level. The majority of respondents (95.44%) reported no income-generating activity.

Respondents were almost equally distributed between Fulgazi (50.60%) and Sonagazi (49.40%) upazilas. All respondents lived with their families, and 89.48% reported the presence of at least one vulnerable household member, including children, older adults, pregnant women, or persons with chronic illnesses. Regarding housing conditions, 44.25% lived in kaccha houses, 30.56% in semi-pucca houses, and 25.20% in pucca houses.

### Flood-related experiences and perceptions

Table 2 presents respondents’ flood-related experiences and perceptions. Most respondents (89.48%) reported no previous flood experience, while 10.52% had experienced flooding previously. The majority (96.23%) perceived their living area as moderately safe from flooding, whereas 3.77% perceived it as unsafe.

**Table 2:** 2024 flash flood-related experiences and perceptions of respondents (*n* = 504).

Feature	Category	*n* (%)
Previous experience of a flood	Yes	53 (10.52)
	No	451 (89.48)
Perceived safety of living place from flood	Moderately safe	485 (96.23)
	Unsafe	19 (3.77)
Received socioeconomic support during the 2024 flash flood	Yes	221 (43.85)
	No	283 (56.15)
Received an early warning during the 2024 flash flood	No	504 (100.00)
Rating of the early warning mechanism	Insufficient	40 (7.94)
	No early warning dissemination mechanism at all	464 (92.06)
The house was inundated during the 2024 flash flood	Yes	504 (100.00)
Duration of the 2024 flash flood	4–6 days	6 (1.19)
	7–10 days	496 (98.41)
	≥11 days	2 (0.40)
Access to safe drinking water during the 2024 flash flood	Yes	42 (8.33)
	No	462 (91.67)
Food scarcity during the 2024 flash flood	Yes	393 (77.98)
	No	111 (22.02)
Evacuated to a shelter during the 2024 flash flood	Yes	376 (74.60)
	No	128 (25.40)
Lack of capacity to protect from flood exposure	Agree	502 (99.60)
	Neutral	2 (0.40)

All respondents (100.00%) reported household inundation during the flood. More than half (56.15%) reported receiving no socioeconomic support during the flood period. No participant reported receiving an early warning before the flood. Regarding local warning dissemination, 92.06% reported that no early warning dissemination mechanism was available in their locality, while 7.94% reported that the available mechanism was insufficient. Most respondents (98.41%) reported a flood duration of 7–10 days. Access to safe drinking water was unavailable for 91.67% of respondents, and 77.98% experienced food scarcity. Shelter evacuation was reported by 74.60% of respondents.

### Damage and loss

Table 3 summarizes damage, loss, and health impacts associated with the 2024 flash flood. All respondents (100.00%) reported that their family income was affected by the flood. No respondents reported loss of family members or injury among family members. Personal injury was reported by 0.79% of respondents, while 7.14% reported illness following the flood. Additionally, 24.40% reported that at least one family member experienced illness during the post-flood period. All respondents reported experiencing mental health impacts related to the flood. Because responses to this variable showed no variation, it was retained only as a descriptive indicator and was not included in inferential analyses.

**Table 3:** Damage, loss, and health impacts due to the 2024 flash flood (*n* = 504).

Feature	Category	*n* (%)
Family income affected by the 2024 flash flood	Yes	504 (100.00)
Loss of a family member due to the 2024 flash flood	No	504 (100.00)
Mental health impact due to the 2024 flash flood	Yes	504 (100.00)
Personal injury due to the 2024 flash flood	Yes	4 (0.79)
	No	500 (99.21)
Disease due to the 2024 flash flood	Yes	36 (7.14)
	No	468 (92.86)
Family member injured during the 2024 flash flood	No	504 (100.00)
Family member affected by the disease during the 2024 flash flood	Yes	123 (24.40)
	No	381 (75.60)

### Severity of depression, anxiety, and stress

**Figure 3:**
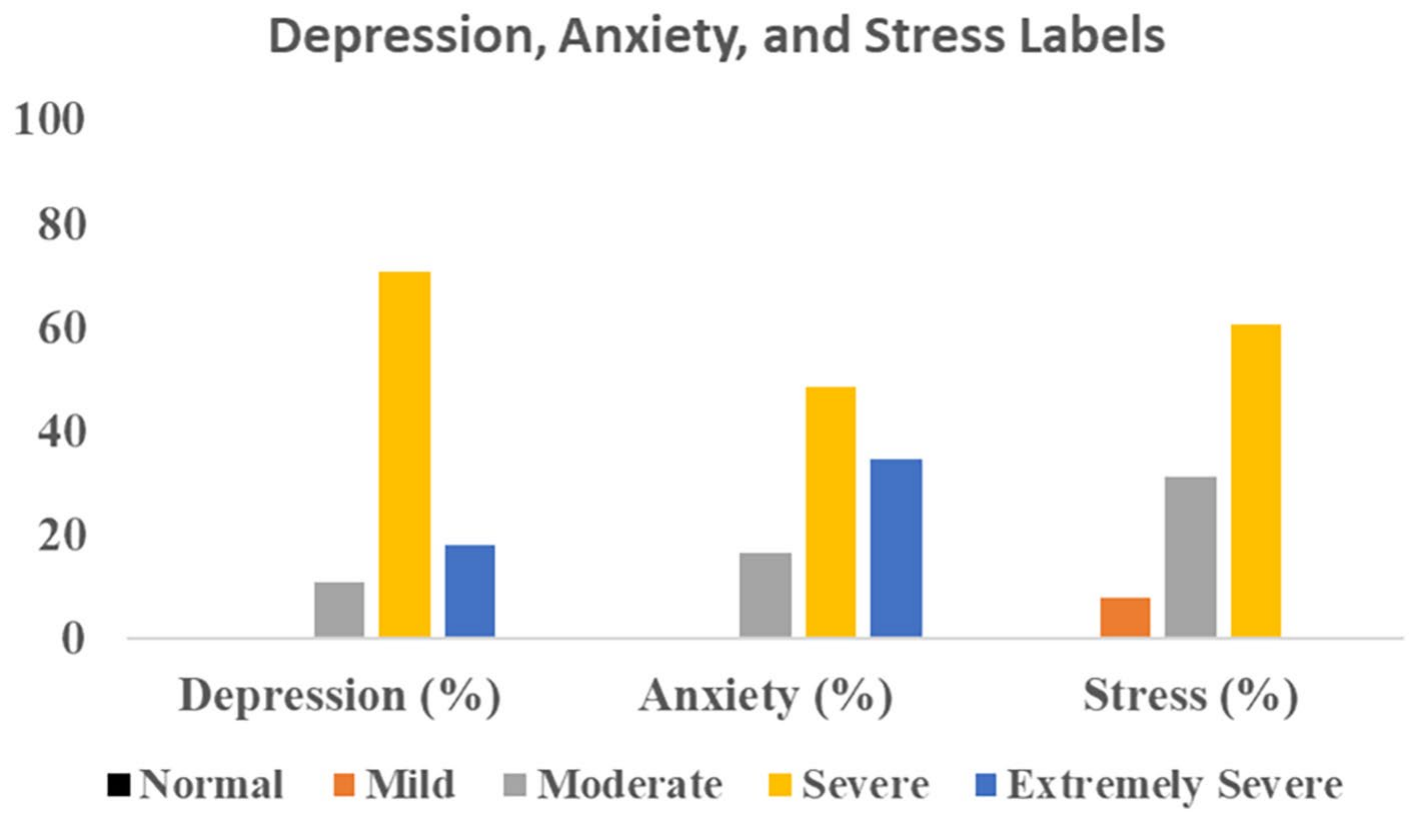
Depression, anxiety, and stress labels among respondents.

**Figure 4:**
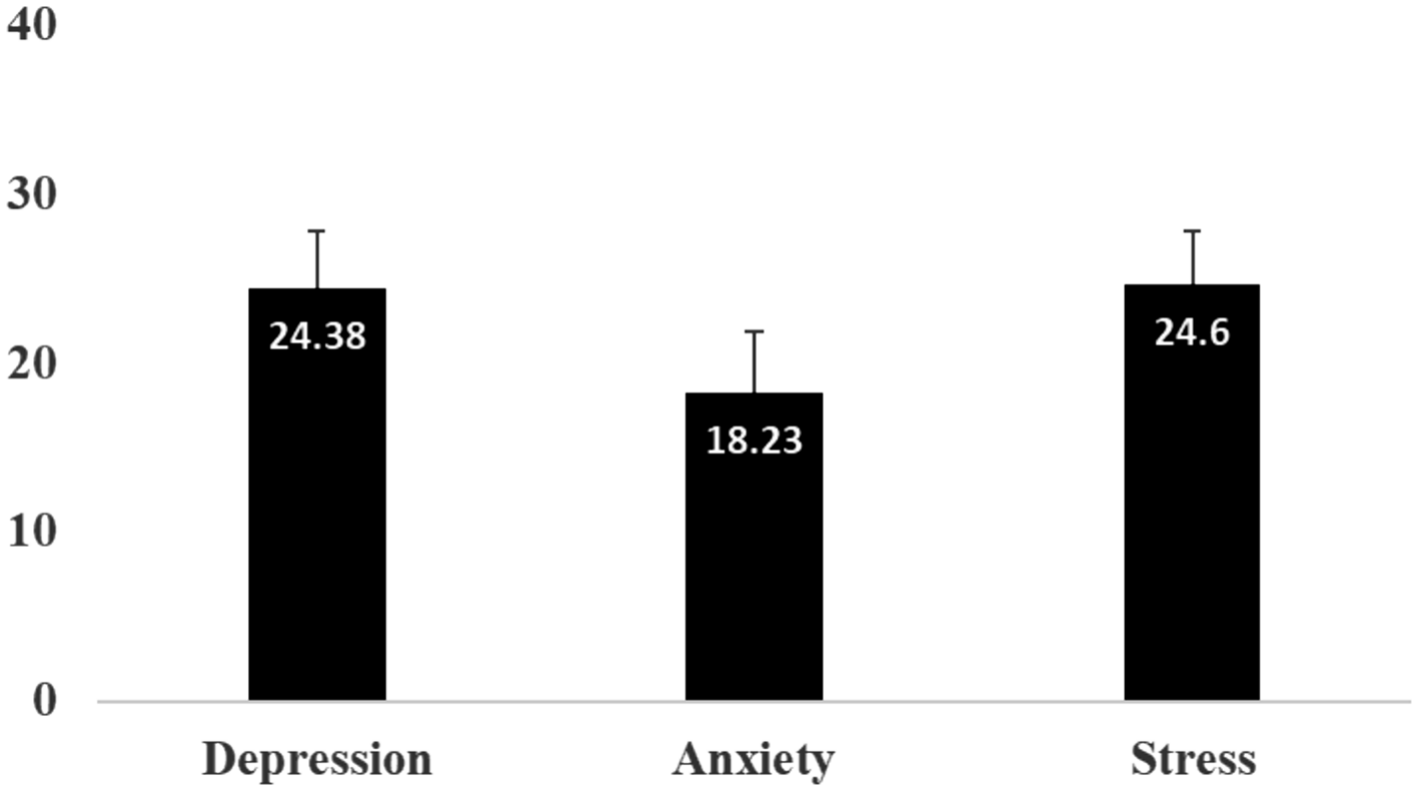
Mean and standard deviation of depression, anxiety, and stress scores among respondents.

Figure 3 presents the severity distribution of depression, anxiety, and stress symptoms measured using the DASS-21. For depression, 71.1% of respondents were classified as severe and 18.1% as extremely severe, while 10.9% were classified as moderate. No respondents were classified as normal or mild. For anxiety, 51.2% of respondents were classified as severe, 16.3% as extremely severe, and 32.5% as moderate. No respondents were classified as normal or mild. For stress, 60.6% of respondents were classified as severe, 31.2% as moderate, 7.9% as mild, and 0.4% as normal. No respondents were classified as extremely severe. Figure 4 presents the mean and standard deviation of depression, anxiety, and stress scores among respondents.

### Factors associated with depression, anxiety, and stress

Table 4 presents the results of the multiple linear regression analyses examining factors associated with depression, anxiety, and stress. Place of residence was significantly associated with all three outcomes. Compared with respondents residing in Fulgazi, those residing in Sonagazi reported higher depression, anxiety, and stress scores. Perceived social life satisfaction was significantly associated with depression, anxiety, and stress. Respondents reporting greater social life satisfaction generally reported lower symptom scores. Income-generating activity was significantly associated with all three mental health outcomes. Respondents engaged in income-generating activities reported lower depression, anxiety, and stress scores than unemployed respondents. Several disaster-related variables were also associated with mental health outcomes, including perceived safety of residence, socioeconomic support, access to safe drinking water, food scarcity, evacuation experience, previous flood experience, flood duration, chronic disease status, and flood-related illness. Because some categories contained small numbers of respondents, particularly respondents aged 18–25 years, those reporting chronic disease, those engaged in income-generating work, and those reporting a flood duration of 11 days or longer, findings related to these categories should be interpreted cautiously.

**Table 4:** Multiple linear regression results for depression (Model I), anxiety (Model II), and stress (Model III).

Features	B coefficient (95% CI)		
	Model I depression	Model II anxiety	Model III stress
Age			
18–25	−3.45 (−5.63, −1.26)**	−2.33 (−4.26, −0.40)*	−1.06 (−2.90, 0.77)
26–35	−0.39 (−1.19, 0.40)	−0.33 (−1.03, 0.38)	−0.36 (−1.03, 0.31)
36–45	Reference	Reference	Reference
46–55	**−0.25 (−0.84, 0.34)**	0.51 (−0.01, 1.04)	0.28 (−0.22, 0.78)
>55	−0.87 (−1.71, −0.03)*	1.36 (0.62, 2.10)***	0.14 (−0.57, 0.84)
Highest education			
Illiterate	0.84 (−0.49, 2.17)	0.32 (−0.86, 1.49)	0.29 (−0.83, 1.41)
Non-SSC	Reference	Reference	Reference
SSC	−0.59 (−1.18, 0.01)	0.07 (−0.45, 0.60)	−0.35 (−0.85, 0.15)
More than SSC	0.96 (−0.24, 2.16)	0.46 (−0.60, 1.52)	0.25 (−0.76, 1.26)
Marital status			
Married	Reference	Reference	Reference
Other (separated/divorced/widowed)	−0.06 (−2.04, 1.91)	0.09 (−1.66, 1.83)	0.00 (−1.66, 1.66)
Occupation			
Some income-generating work	−2.68 (−3.89, −1.47)***	−1.64 (−2.71, −0.57)**	−3.31 (−4.32, −2.29)***
Unemployed	Reference	Reference	Reference
Types of household			
Kaccha	Reference	Reference	Reference
Semi-Pucca	−0.44 (−1.00, 0.13)	−0.36 (−0.86, 0.14)	−0.15 (−0.62, 0.33)
Pucca	−0.88 (−2.20, 0.43)	0.45 (−0.71, 1.62)	0.66 (−0.44, 1.77)
Presence of vulnerable people in family			
Yes	Reference	Reference	Reference
No	2.48 (1.37, 3.59)***	0.65 (−0.33, 1.63)	2.18 (1.25, 3.11)***
Chronic diseases			
Yes	−1.40 (−3.70, 0.89)	7.66 (5.63, 9.69)***	−0.56 (−2.49, 1.37)
No	Reference	Reference	Reference
Maybe	−3.89 (−5.49, −2.28)***	−3.47 (−4.89, −2.05)***	−3.95 (−5.30, −2.60)***
Name of the Upazila			
Fulgazi	Reference	Reference	Reference
Sonagazi	0.96 (0.38, 1.53)**	1.17 (0.66, 1.68)***	1.94 (1.46, 2.43)***
Perception of current social life			
Least satisfied	2.38 (1.09, 3.66)***	1.24 (0.10, 2.37)*	1.65 (0.56, 2.73)**
Satisfied	Reference	Reference	Reference
Very satisfied	−5.58 (−6.97, −4.19)***	−3.78 (−5.01, −2.54)***	−5.69 (−6.86, −4.52)***
Perceived safety from flood			
Moderately safe	Reference	Reference	Reference
Unsafe	0.96 (−0.37, 2.29)	2.64 (1.47, 3.82)***	1.07 (−0.05, 2.19)
Previous flood experience			
Yes	−0.74 (−1.55, 0.08)	−0.32 (−1.04, 0.40)	−1.14 (−1.83, −0.45)**
No	Reference	Reference	Reference
Flood duration			
4–6 days	1.84 (−0.64, 4.33)	0.62 (−1.57, 2.81)	3.52 (1.44, 5.61)***
7–10 days	Reference	Reference	Reference
≥11 days	−1.28 (−5.10, 2.54)	−7.16 (−10.54, −3.79)***	−2.44 (−5.64, 0.77)
Socioeconomic support during flood			
Yes	−0.30 (−0.88, 0.28)	−0.82 (−1.33, −0.31)**	−0.93 (−1.42, −0.44)***
No	Reference	Reference	Reference
Early warning mechanism rating			
Insufficient	2.51 (1.16, 3.85)***	6.70 (5.52, 7.89)***	−1.76 (−2.89, −0.63)**
No early warning dissemination mechanism at all	Reference	Reference	Reference
Access to safe drinking water			
Yes	0.88 (−0.65, 2.40)	−3.03 (−4.38, −1.68)***	0.52 (−0.76, 1.80)
No	Reference	Reference	Reference
Food scarcity during flood			
Yes	Reference	Reference	Reference
No	−1.83 (−2.65, −1.02)***	0.73 (0.01, 1.45)*	−0.47 (−1.16, 0.21)
Evacuation to shelter			
Yes	Reference	Reference	Reference
No	1.40 (0.12, 2.68)*	−1.35 (−2.48, −0.22)*	−0.54 (−1.62, 0.53)
Disease due to flood			
Yes	2.68 (1.31, 4.05)***	1.99 (0.78, 3.20)**	3.77 (2.62, 4.93)***
No	Reference	Reference	Reference
Family member disease			
Yes	1.00 (0.37, 1.63)**	−0.15 (−0.70, 0.41)	0.20 (−0.33, 0.74)
No	Reference	Reference	Reference
R^2^	0.485	0.653	0.596
Adjusted R^2^	0.455	0.633	0.572

*p < 0.05; **p < 0.01; ***p < 0.001; CI, confidence interval. Bold shows the significant value.

## Discussion

This study examined the prevalence and associated factors of depression, anxiety, and stress among women affected by the 2024 flash flood in Feni District, Bangladesh. The findings revealed a substantial burden of psychological distress, with most respondents reporting severe or extremely severe symptoms of depression, anxiety, and stress. Mental health outcomes were associated with place of residence, social life satisfaction, income-generating activity, perceived safety, chronic disease, flood-related illness, socioeconomic support, and access to essential resources.

The high prevalence of psychological distress observed in this study is consistent with previous research demonstrating elevated levels of depression, anxiety, and stress among populations exposed to disasters and flooding (6, 7, 16). Similar findings have been reported among flood-affected populations in Bangladesh and other disaster-prone settings, where prolonged disruption of livelihoods, displacement, uncertainty, and loss of resources contribute to adverse psychological outcomes.

Women may be particularly vulnerable to post-disaster psychological distress because of gender-specific social roles and responsibilities. Consistent with the Gender and Disaster Vulnerability Framework, women frequently bear primary responsibility for childcare, household management, food preparation, water collection, and caregiving for vulnerable family members. During disasters, these responsibilities often intensify while access to resources becomes more constrained. Economic dependence, caregiving burdens, reduced mobility, and limited access to assistance may therefore contribute to elevated levels of depression, anxiety, and stress among women following floods (18–20).

One of the most consistent findings of this study was the association between higher social life satisfaction and lower levels of depression, anxiety, and stress. Importantly, social life satisfaction should be interpreted as perceived social well-being rather than a direct measure of social support because the present study did not employ a validated social support instrument. Nevertheless, women who reported greater satisfaction with their social life may have benefited from stronger social connectedness, a greater sense of belonging, and more positive interpersonal relationships, all of which may facilitate psychological adjustment following disasters. Similarly, women who received socioeconomic support during or after the flood generally reported lower levels of psychological distress. These findings can be interpreted through the lens of psychosocial resources, whereby both social connectedness and material assistance may help individuals cope with adversity, reduce uncertainty, and maintain a sense of security during recovery. Previous disaster research suggests that contextual support from family members, communities, religious leaders, and institutions can play an important role in helping survivors adapt to traumatic experiences, reconstruct meaning, and strengthen resilience during post-disaster recovery processes. Supportive social environments may therefore contribute not only to material recovery but also to emotional adaptation and psychological well-being (35, 36). Furthermore, psychosocial resources have been shown to facilitate recovery by promoting social connectedness, enhancing resilience, and fostering adaptive coping mechanisms among disaster-affected populations (36, 37).

Women engaged in income-generating activities reported lower levels of depression, anxiety, and stress than unemployed women. This finding may reflect the potential role of economic agency, perceived control, financial security, and access to resources in shaping psychological well-being following disasters. However, this finding should be interpreted cautiously because only a small proportion of respondents reported income-generating activities, which may affect the stability of the estimate.

Women residing in Sonagazi reported higher levels of depression, anxiety, and stress than those residing in Fulgazi. However, the present study did not collect detailed information regarding flood intensity, housing damage, recovery resources, or aid distribution across locations. Therefore, the observed geographic variation should be interpreted cautiously and warrants further investigation in future studies.

The findings also highlight the importance of disaster-related exposures and living conditions. Women experiencing chronic illness, flood-related illness, perceived lack of safety, limited socioeconomic support, and restricted access to safe drinking water generally reported poorer mental health outcomes. These findings are consistent with the Conservation of Resources Theory, which suggests that actual or threatened loss of valued resources and ongoing security threats contribute to psychological distress following disasters (21).

Several findings were counterintuitive, including associations involving flood duration, food scarcity, and perceived adequacy of early warning dissemination. Lower anxiety among respondents reporting longer flood duration and mixed associations involving food scarcity should be interpreted as exploratory findings. Because some categories contained very small numbers of respondents, particularly those reporting flood duration of 11 days or longer, these patterns may reflect sampling variability, residual confounding, measurement limitations, or contextual factors rather than true protective effects. Consequently, these associations require validation in future studies using larger and more representative samples.

The findings have several practical implications. Integrating mental health and psychosocial support into disaster preparedness, response, and recovery programs may help address the substantial psychological burden experienced by women following floods. Gender-sensitive interventions that strengthen access to social resources, livelihood opportunities, safe drinking water, healthcare services, and community-based support systems may contribute to improved psychological well-being and disaster resilience.

### Limitations and strengths

This study has several limitations that should be considered when interpreting the findings. First, the cross-sectional design precludes causal inference, and the absence of a non-exposed comparison group limits the ability to determine the extent to which the observed mental health outcomes can be directly attributed to flood exposure. Consequently, the findings reflect associations among flood-affected women rather than causal effects of the disaster. In addition, data were collected at a single point in time, preventing assessment of changes in psychological well-being throughout the recovery process.

Second, the use of purposive sampling may limit the generalizability of the findings to other flood-affected populations. Although this approach facilitated the recruitment of women directly affected by the 2024 flash flood, the sample may not fully represent all women living in flood-prone regions, particularly those who were unavailable during data collection or those experiencing either minimal or extremely severe psychological distress. Furthermore, the exclusion of women with severe illness may have resulted in the underrepresentation of some of the most vulnerable individuals.

Third, all measures relied on self-reported information and may therefore be subject to recall bias, reporting bias, and individual differences in perception. Although a validated Bengali version of the DASS-21 was employed, the instrument assesses the severity of depression, anxiety, and stress symptoms rather than providing clinical diagnoses of mental disorders. Fourth, although a broad range of sociodemographic, health-related, and disaster-related variables was included in the analyses, residual confounding due to unmeasured factors cannot be excluded. In addition, several predictor categories contained relatively small numbers of respondents, particularly younger women, those engaged in income-generating activities, respondents with chronic diseases, and those reporting prolonged flood duration, which may have affected the stability of some estimates.

Finally, the high concentration of respondents within the severe categories of depression, anxiety, and stress should be interpreted cautiously. Although the scoring procedures were verified and the distributions were consistent with the observed data, this pattern may partly reflect contextual factors, including widespread disruption of livelihoods, limited access to services, and the sampling strategy. The possibility of overrepresentation of highly affected individuals cannot be entirely ruled out.

Despite these limitations, the study has several notable strengths. It focuses exclusively on women, enabling a gender-sensitive examination of mental health outcomes in a disaster context. The relatively large sample size enhances statistical reliability and provides one of the few empirical assessments of women’s mental health following the 2024 flash flood in Feni District. The study also contributes timely and context-specific evidence to the growing literature on disaster-related mental health in Bangladesh. Furthermore, the inclusion of a wide range of sociodemographic, health-related, and disaster-exposure variables provides a more comprehensive understanding of factors associated with psychological distress than many previous studies. The use of the standardized Bengali version of the DASS-21 further enhances methodological rigor and facilitates comparison with national and international research.

Future research should employ longitudinal designs, larger and more representative samples, and mixed-method approaches to better understand the persistence, trajectories, and underlying mechanisms of psychological distress among women affected by floods. Further investigation is also needed to examine geographic variations in mental health outcomes and to validate the exploratory associations observed in the present study, particularly those involving flood duration, food scarcity, and early warning dissemination.

### Recommendations

The findings of this study indicate a substantial level of reported psychological distress and highlight the importance of strengthening mental health considerations within disaster management frameworks, particularly for women in flood-prone regions. While causal relationships cannot be established, the observed associations suggest that integrating mental health and psychosocial support into disaster preparedness, response, and recovery may be beneficial. Community-based mental health interventions can be incorporated into post-disaster relief efforts, with a focus on screening, referral, and supportive care for women reporting elevated levels of depression, anxiety, and stress. Training local health workers and community volunteers to recognize psychological distress and provide basic psychosocial support may help address existing service gaps in resource-constrained settings.

Strengthening social support networks and community cohesion should be prioritized, as perceived social life satisfaction was consistently associated with lower psychological distress. Programs that promote women’s social participation, peer support groups, and community engagement may enhance coping capacity during post-disaster recovery. Similarly, the association between unemployment and greater distress suggests that economic empowerment initiatives, such as livelihood restoration, cash-for-work programs, and skills development, may support improved well-being. However, further research is needed to confirm these relationships.

Improving disaster preparedness remains essential. It includes strengthening early warning dissemination systems, enhancing risk communication, and ensuring equitable access to socioeconomic support during floods. However, given some mixed and counterintuitive findings (e.g., early warning and flood duration), these aspects should be interpreted cautiously and warrant further investigation before drawing firm programmatic conclusions. Ensuring access to basic services such as safe drinking water, adequate food supplies, and gender-sensitive shelter facilities is also important, as these factors were associated with psychological outcomes in this study. Policymakers are encouraged to adopt gender-responsive, evidence-informed disaster risk reduction strategies that incorporate mental health considerations into broader climate adaptation and resilience planning in Bangladesh.

## Conclusion

This study provides context-specific evidence on the distribution and associated factors of depression, anxiety, and stress among women affected by the 2024 flash flood in Feni, Bangladesh. A high concentration of respondents reported symptoms in moderate to extremely severe categories. However, these findings should be interpreted as reflecting the distribution of psychological distress within a purposively selected, flood-affected sample rather than population-level prevalence or causal effects of the flood. The results suggest that women’s mental health outcomes were associated with a combination of geographic location, social life satisfaction, employment status, perceived safety, health conditions, and disaster-related experiences, highlighting the multidimensional nature of post-disaster psychological well-being. Rather than indicating direct effects, these associations point to intersecting social, economic, and environmental vulnerabilities that may shape mental health in disaster contexts. Overall, the study underscores that mental health is an important component of disaster impact and recovery, particularly for women who often carry significant caregiving and household responsibilities. Strengthening access to psychosocial support, improving basic service delivery, and enhancing preparedness mechanisms may improve recovery experiences. At the same time, further longitudinal and comparative research is needed to better understand the magnitude, persistence, and causal pathways of mental health outcomes in flood-affected populations. Recognizing and addressing women’s mental health within disaster response and resilience planning is therefore an important consideration for sustainable recovery in Bangladesh.

## Data Availability

The raw data supporting the conclusions of this article will be made available by the authors, without undue reservation.
